# Pan-Immune-Inflammatory Value is Superior to Other Inflammatory Indicators in Predicting Inpatient Major Adverse Cardiovascular Events and Severe Coronary Artery Stenosis after Percutaneous Coronary Intervention in STEMI Patients

**DOI:** 10.31083/j.rcm2508294

**Published:** 2024-08-20

**Authors:** Li Yang, Jiongchao Guo, Min Chen, Yuqi Wang, Jun Li, Jing Zhang

**Affiliations:** ^1^Department of Cardiology, The Second People's Hospital of Hefei, Hefei Hospital Affiliated to Anhui Medical University, 230011 Hefei, Anhui, China; ^2^The Fifth Clinical School of Medicine, Anhui Medical University, 230000 Hefei, Anhui, China; ^3^Department of Cardiology, Anhui Medical University Third Affiliated Hospital, 230011 Hefei, Anhui, China; ^4^Department of Cardiology, Hefei Second People's Hospital Affiliated to Bengbu Medical College, 230011 Hefei, Anhui, China; ^5^Department of Cardiology, Lu'an Municipal People's Hospital, 230011 Hefei, Anhui, China

**Keywords:** pan-immune-inflammation value, systemic immune-inflammation, platelet-to-lymphocyte ratio, neutrophil-to-lymphocyte ratio, acute myocardial infarction, coronary artery disease

## Abstract

**Background:**

The inflammatory response to atherosclerosis is a process that leads to coronary artery disease. Pan-immune-inflammation value (PIV) has emerged as a new and simple biomarker of inflammation. However, studies on the predictive power of PIV for major adverse cardiovascular events (MACE) or the degree of coronary artery stenosis are scarce. We aimed to explore the predictive ability of PIV for MACE and the degree of coronary artery stenosis in patients with ST-segment elevation myocardial infarction (STEMI) after percutaneous coronary intervention (PCI) during hospitalization.

**Methods:**

This study included 542 patients who were diagnosed with STEMI and who underwent PCI between 2016 and 2023 and whose PIV and other inflammatory markers were measured. Using univariate and multivariate logistic regression analysis, risk variables for MACE following PCI and severe coronary stenosis during hospitalization were assessed to create receiver operating characteristic (ROC) curves and determine the best thresholds for inflammatory markers. Spearman correlation analysis was used to evaluate the correlation of PIV and other inflammatory markers with the Gensini score (GS).

**Results:**

Compared with the systemic inflammatory index (SII), platelet-to-lymphocyte ratio (PLR), and neutrophil-to-lymphocyte ratio (NLR), the PIV may have greater predictive value in terms of the occurrence of MACE and the degree of coronary stenosis after PCI in hospitalized STEMI patients. The correlation between the PIV and GS was strong.

**Conclusions:**

PIV was superior to the SII, PLR, and NLR in predicting inpatient prognosis and severe coronary stenosis after PCI for STEMI patients.

## 1. Introduction

Cardiovascular disease (CVD) is one of the leading causes of death. It is estimated that approximately 127.9 million (48.6%) Americans aged 20 and older have CVD, and the overall prevalence of CVD excluding hypertension (coronary atherosclerotic heart disease, heart failure, and stroke only) is 9.9% (28.6 million in 2020). According to the National Health and Nutrition Examination Survey (NHANES) 2017–2020, in the United States (US), the overall incidence of acute myocardial infarction (AMI), a subtype of coronary artery disease (CAD), is 3.2% in adults [1]. Despite advances in antithrombotic therapy and reperfusion techniques and a significant decrease in AMI mortality, the prognosis remains poor [2]. Therefore, early identification and intervention are crucial.

Atherosclerosis, the leading cause of AMI, was previously thought to be simply a disease in which cholesterol builds up in the walls of blood vessels [3, 4, 5, 6, 7]. The systemic inflammatory index (SII), the neutrophil-to-lymphocyte ratio (NLR), and the platelet-to-lymphocyte ratio (PLR) are widely used inflammatory parameters [8, 9, 10, 11, 12]. They are calculated using peripheral blood biomarkers such as platelets, neutrophils, monocytes, and lymphocytes, as well as other peripheral blood biomarkers [13]. NLR and PLR predict the prognosis of patients with ST-segment elevation myocardial infarction (STEMI) undergoing percutaneous coronary intervention (PCI) [14]. SII, NLR, and PLR predict the clinical prognosis of patients with acute coronary syndrome (ACS), and their levels correlate closely with major adverse cardiovascular events (MACE) in patients with STEMI [13, 14, 15]. A new parameter, the pan-immune-inflammation value (PIV), which considers all blood cell populations (monocytes × neutrophils × platelets/lymphocytes) reflecting systemic inflammation and immunity, has been shown in several studies to be significantly correlated with the clinical prognosis of cancer patients [16, 17, 18]. It is worth noting that PIV, as a new comprehensive index, has a predictive value for blood flow in STEMI patients [19, 20, 21]. However, studies related to the predictive value of PIV with other inflammatory indexes for MACE after PCI and the degree of coronary artery stenosis in STEMI patients have not been conducted.

The purpose of this study was to further evaluate the predictive value of the PIV for the degree of coronary stenosis as determined by the Gensini score (GS) and to explore the predictive value of the PIV, SII, NLR, and PLR for the occurrence of MACE following PCI in patients hospitalized with STEMI.

## 2. Materials and Methods

### 2.1 Study Design and Patient Inclusion-exclusion Criteria

**Fig. 1.**
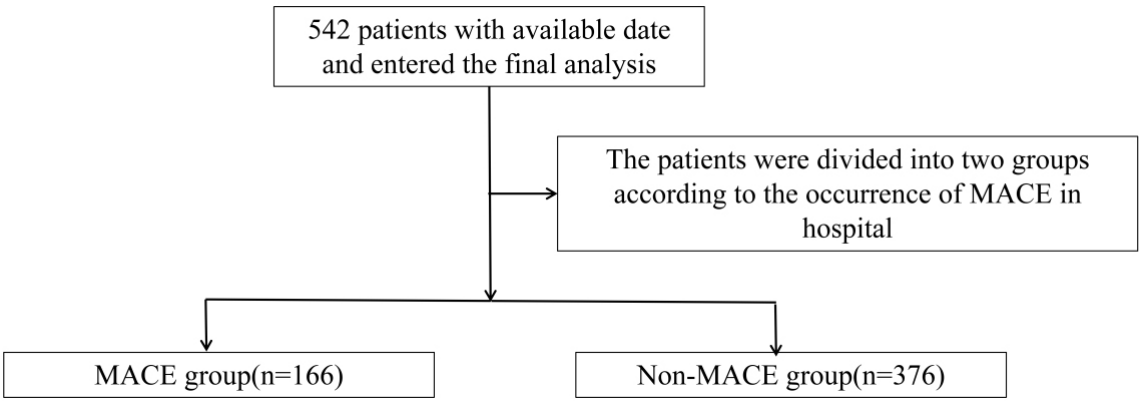
**Select flowchart**. MACE, major adverse cardiovascular event.

This was a single-center retrospective study. The study included 542 patients who were diagnosed with STEMI and underwent PCI between 2016 and 2023 at Hefei Hospital Affiliated to Anhui Medical University (Fig. 1). The inclusion criteria were as follows: (1) Patients with a definite diagnosis of AMI. (2) All patients signed informed consent and underwent coronary angiography (CAG). (3) Successful recanalization of the occluded vessel by initial PCI. Exclusion criteria: (1) Patients who lacked critical baseline data. (2) Patients who refused to undergo CAG or for other reasons, the extent of coronary artery disease was unknown. (3) Patients with severe hepatic or renal abnormalities, autoimmune disorders, hematological disorders (e.g., leukemia, aplastic anemia), malignant tumors, or recent severe infections. (4) Patients with previous PCI or coronary bypass grafting treatment. (5) Patients in whom initial PCI failed to recanalize the offending vessel. Ultimately, a total of 542 patients were included in this study.

### 2.2 Data Collection

The information included in this study was: (1) Clinical information: age, gender, smoking, hypertension, diabetes, Killip classification. (2) Vital sign information: systolic blood pressure (SP), diastolic blood pressure (DP), and heart rate (HR). (3) Ancillary examination information (all blood samples were collected within 24 hours of admission to the hospital): neutrophils, monocytes, lymphocytes, platelets, serum creatinine (sCr), blood urea nitrogen (BUN), glomerular filtration rate (eGFR), uric acid (UA), total protein (TP), triglycerides (TG), total cholesterol (TC), left ventricular ejection fraction (LVEF), and left ventricular fractional shortening (LVFS). (4) CAG-related data: coronary stenosis degree.

### 2.3 Definitions

The following definitions were utilized in this study:

(1) MACE during hospitalization: includes all-cause death (death from any cause) during hospitalization with 1-week follow-up, new heart failure after AMI (new heart failure after this infarction in a patient with no previous history of heart failure, diagnosed primarily based on clinical symptoms, such as paroxysmal nocturnal dyspnea, the presence of rales in the lungs, the presence of symptoms of acute pulmonary edema, upper and lower extremity edema at the ankles, pleural effusion, and BNP and cardiac ultrasound), new stroke after AMI, recurrent AMI, and malignant arrhythmias (including ventricular tachycardia, ventricular fibrillation, third-degree atrioventricular block, and other life-threatening arrhythmias that lead to hemodynamic instability in patients).

(2) PIV was defined as (monocytes × neutrophils × platelets)/lymphocytes, SII as (platelets × neutrophils)/lymphocytes, PLR as platelets/lymphocytes, and NLR as neutrophils/lymphocytes.

(3) The Gensini scoring system is widely used for evaluating the severity of coronary artery lesions. GS was categorized into group 1 (GS <44), group 2 (44 ≤ GS ≤ 80), and group 3 (GS >80) based on tertiles. GS was categorized into group 1 (GS <44) with 146 patients; group 2 (44 ≤ GS ≤ 80) with 218 patients; and group 3 (GS >80) with 178 patients based on tertiles.

### 2.4 Statistical Analysis

Statistical analyses were performed using IBM SPSS Statistics 27.0 (IBM, Armonk, NY, USA), GraphPad Prism software (version 10.1.2, GraphPad Prism Software Inc., San Diego, CA, USA), and R studio (version 4.3.1, Chinese Academy of Sciences, Beijing, China). Normally distributed continuous variables were expressed as mean ± standard deviation and Student’s *t*-test was used to compare differences between groups. Continuous variables with skewed distributions were expressed as median M (P25, P75), and the Mann-Whitney U test or Kruskal-Wallis H test was used to compare differences between groups. Categorical variables were expressed as frequencies (percentages) and differences between groups were compared using the Chi-square test or Fisher’s test. One-way analysis of variance (ANOVA) was used to evaluate GS data that were normally distributed among the three groups. Logistic regression analysis was used to evaluate the risk factors. The risk of MACE and the severity of coronary stenosis in various subgroups were examined using subgroup analysis. The critical levels and predictive power of the PIV, SII, PLR, and NLR were ascertained using receiver operating characteristic (ROC) curves. The optimal thresholds, sensitivity, specificity, and 95% confidence intervals (CIs) were calculated. Spearman correlation analysis was used to assess the correlation between the PIV, SII, PLR, NLR, and GS parameters. In addition, two-sided *p* values < 0.05 were statistically significant.

## 3. Results

### 3.1 Baseline Characteristics

**Fig. 2.**
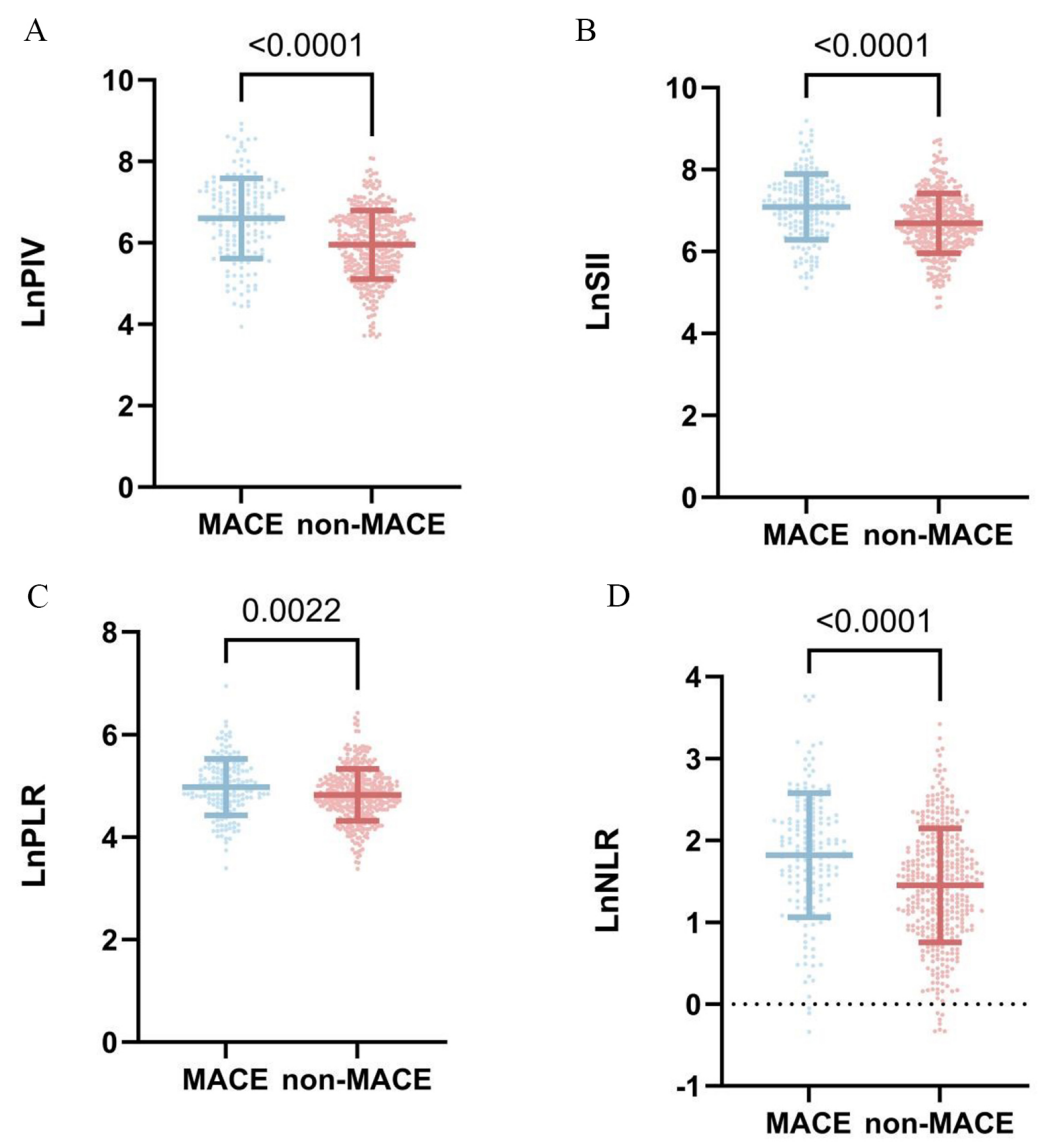
**Comparison of inflammatory markers for MACE in-hospital**. (A) LnPIV levels between MACE and Non-MACE. (B) LnSII levels between MACE and Non-MACE. (C) LnPLR levels between MACE and Non-MACE. (D) LnNLR levels between MACE and Non-MACE. LnPIV, natural logarithmic transformation of pan-immune-inflammation values; LnSII, natural logarithmic transformation of systemic immune-inflammation index; LnPLR, natural logarithmic transformation of platelet-to-lymphocyte ratio; LnNLR, natural logarithmic transformation of neutrophil-to-lymphocyte ratio; MACE, major adverse cardiovascular events.

A total of 542 patients with AMI were ultimately included in this study, with 166 patients (16 deaths, 69 post-infarction heart failures, 64 arrhythmias, 17 recurrent infarctions) in the MACE group and 376 patients in the non-MACE group. In the comparison of baseline data, age, DP, HR, neutrophils, monocytes, natural logarithmic transformation of PIV (LnPIV), the natural logarithmic transformation of the SII (LnSII), natural logarithmic transformation of PLR (LnPLR), natural logarithmic transformation of NLR (LnNLR), BUN, sCr, UA, eGFR, TC, GS, LVFS, and Killip Class 2–4 were significantly greater in the MACE group than in the non-MACE group (*p* < 0.05). There was no statistically significant difference in sex, smoking history, history of hypertension, history of diabetes mellitus, SP, lymphocyte count, platelet count, TP, TG, or LVEF between the patients in the MACE group and the patients in the non-MACE group (*p* > 0.05) (Table 1) (Fig. 2).

**Table 1.** **Baseline characteristics of patients with major and non-major adverse cardiovascular events**.

Variables	MACE group (n = 166)	Non-MACE group (n = 376)	*p* value
Age (year)	64.13 ± 14.42	59.73 ± 13.29	<0.001
Male [n (%)]	130 (78.3%)	305 (81.1%)	0.450
Smoking [n (%)]	86 (51.8%)	215 (57.2%)	0.246
Hypertension [n (%)]	104 (62.7%)	206 (54.8%)	0.088
Diabetes [n (%)]	48 (28.9%)	95 (25.3%)	0.374
SP (mmHg)	124.92 ± 26.54	128.28 ± 23.00	0.136
DP (mmHg)	75.03 ± 16.01	78.12 ± 15.70	0.036
HR	81.13 ± 22.02	77.43 ± 15.04	0.023
Neutrophil (×10^9^/L)	8.48 (6.14, 11.28)	6.64 (4.93, 8.79)	<0.001
Lymphocyte (×10^9^/L)	1.30 (0.98, 1.84)	1.52 (1.08, 2.14)	0.013
Monocyte (×10^9^/L)	0.60 (0.42, 0.88)	0.50 (0.39, 0.70)	<0.001
Platelet (×10^9^/L)	195.00 (157.00, 241.25)	196.00 (153.25, 236.00)	0.421
LnPIV	6.60 ± 0.98	5.95 ± 0.84	<0.001
LnSII	7.09 ± 0.80	6.69 ± 0.73	<0.001
LnPLR	4.97 ± 0.55	4.82 ± 0.51	0.002
LnNLR	1.82 ± 0.76	1.45 ± 0.70	<0.001
BUN (mmol/L)	6.35 (4.91, 8.06)	5.30 (4.33, 6.39)	<0.001
Creatinine (μmol/L)	76.00 (61.95, 99.08)	70.80 (58.50, 79.25)	<0.001
eGFR (mL/(min × 1.73 m^2^))	90.49 (66.66, 101.13)	97.53 (83.62, 106.78)	<0.001
UA (mmol/L)	361.46 (305.00, 433.10)	347.80 (289.05, 405.85)	0.027
TP (g/L)	62.95 (59.60, 66.10)	62.85 (59.73, 66.58)	0.709
TG (mmol/L)	1.43 (0.95, 2.05)	1.52 (1.08, 2.27)	0.053
TC (mmol/L)	4.16 (3.55, 4.82)	4.41 (3.80, 5.11)	0.008
Gensini Score	70.00 (44.00, 93.00)	62.00 (42.00, 84.00)	0.021
LVEF	58.90 (52.00, 62.00)	59.00 (56.00, 63.75)	0.005
LVFS	30.40 (28.00, 32.00)	30.40 (29.00, 33.00)	0.020
Killip Class 2–4 [n (%)]	80 (48.2%)	78 (20.7%)	<0.001

Continuous variables are expressed as the mean ± SD, and categorical variables are expressed as percentages. The PIV, SII, PLR, and NLR are skewed data, and natural logarithmic transformations are normally distributed data. SP, systolic blood pressure; DP, diastolic blood pressure; HR, heart rate; SD, standard deviation; MACE, major adverse cardiovascular event; LnPIV, natural logarithmic transformation of pan-immune-inflammation values; LnSII, natural logarithmic transformation of systemic immune-inflammation index; LnPLR, natural logarithmic transformation of platelet-to-lymphocyte ratio; LnNLR, natural logarithmic transformation of neutrophil-to-lymphocyte ratio; BUN, blood urea nitrogen; eGFR, glomerular filtration rate; UA, uric acid; TP, total protein; TG, triglyceride; TC, cholesterol; LVEF, left ventricular ejection fraction; LVFS, left ventricular fractional shortening.

GS was categorized into group 1 (GS <44) with 146 patients; group 2 (44 ≤ GS ≤ 80) with 218 patients; and group 3 (GS >80) with 178 patients based on tertiles. Age, DP, HR, neutrophil count, monocyte count, LnPIV, LnSII, LnPLR, LnNLR, BUN, sCr, UA, eGFR, TC, and Killip class 2–4 were significantly different (*p* < 0.05). The differences in the other indicators were not statistically significant (*p* > 0.05) (Table 2).

**Table 2.** **Baseline characteristics of patients with low (Group 1: GS <44), medium (Group 2: 44 ≤ GS ≤ 80) and high (Group 3: GS >80) Gensini scores**.

Variables	Group 1 (n = 146)	Group 2 (n = 218)	Group 3 (n = 178)	*p*	*p_1⁢–⁢2_*	*p_1⁢–⁢3_*	*p_2⁢–⁢3_*
Age (year)	59.49 ± 12.74	60.47 ± 14.55	63.13 ± 13.47	0.042	0.504	0.018	0.055
Male [n (%)]	122 (83.6%)	170 (78.0%)	143 (80.3%)	0.423			
Smoking [n (%)]	85 (58.2%)	117 (53.7%)	99 (55.6%)	0.693			
Hypertension [n (%)]	88 (60.3%)	121 (55.5%)	101 (56.7%)	0.659			
Diabetes [n (%)]	33 (22.6%)	59 (27.1%)	51 (28.7%)	0.450			
SP (mmHg)	125.84 ± 24.87	126.45 ± 23.77	129.40 ± 24.04	0.342			
DP (mmHg)	76.45 ± 15.85	75.72 ± 15.93	79.54 ± 15.54	0.047	0.663	0.080	0.017
HR	76.44 ± 16.46	77.00 ± 15.47	82.21 ± 20.13	0.003	0.765	0.003	0.003
Neutrophil (×10^9^/L)	6.13 (4.25, 8.61)	6.98 (5.23, 9.04)	7.90 (5.77, 10.82)	<0.001	0.023	<0.001	0.004
Lymphocyte (×10^9^/L)	1.60 (1.18, 2.26)	1.42 (1.00, 2.09)	1.36 (1.05, 1.89)	0.022	0.072	0.028	1.000
Monocyte (×10^9^/L)	0.51 (0.40, 0.70)	0.50 (0.38, 0.70)	0.60 (0.40, 0.80)	0.005	0.566	0.280	0.004
Platelet (×10^9^/L)	196.00 (148.75, 240.00)	197.50 (155.00, 242.00)	189.00 (157.00, 230.75)	0.602			
LnPIV	5.32 ± 0.53	6.50 ± 0.60	6.41 ± 1.09	<0.001	<0.001	<0.001	0.242
LnSII	6.23 ± 0.55	7.06 ± 0.61	6.99 ± 0.86	<0.001	<0.001	<0.001	0.315
LnPLR	4.59 ± 0.44	4.98 ± 0.49	4.97 ± 0.55	<0.001	<0.001	<0.001	0.824
LnNLR	1.04 ± 0.60	1.77 ± 0.61	1.74 ± 0.77	<0.001	<0.001	<0.001	0.707
BUN (mmol/L)	5.53 (4.34, 6.83)	5.34 (4.33, 6.55)	5.93 (4.79, 7.36)	<0.002	1.000	0.032	0.003
Creatinine (μmol/L)	71.10 (60.00, 81.55)	71.00 (58.58, 81.10)	72.40 (60.93, 88.00)	0.381			
eGFR (mL/(min × 1.73 m^2^))	97.09 (82.80, 103.94)	92.80 (83.55, 106.87)	93.25 (72.59, 105.20)	0.327			
UA (mmol/L)	361.46 (294.80, 415.00)	334.50 (287.83, 402.70)	361.46 (295.40, 428.53)	0.065			
TP (g/L)	62.35 (59.20, 65.60)	62.70 (59.30, 66.30)	63.13 (60.48, 67.30)	0.097			
TG (mmol/L)	1.51 (1.10, 2.23)	1.52 (1.04, 2.20)	1.49 (1.06, 2.10)	0.879			
TC (mmol/L)	4.23 (3.55, 4.84)	4.25 (3.66, 5.08)	4.42 (3.88, 5.28)	0.006	1.000	0.008	0.038
LVEF	59.00 (57.00, 64.00)	58.90 (55.00, 63.25)	58.90 (53.75, 62.00)	0.147			
LVFS	30.40 (30.00, 33.25)	30.40 (29.00, 33.00)	30.40 (29.00, 33.00)	0.502			
Killip Class 2–4 [n (%)]	44 (30.1%)	48 (22.0%)	66 (37.1%)	0.004	0.081	0.189	<0.001

Continuous variables are expressed as the mean ± SD, and categorical variables are expressed as percentages. The PIV, SII, PLR, and NLR are skewed data, and the natural logarithmic transformation is used for normally distributed data. SD, standard deviation; GS, Gensini score; LnPIV, natural logarithmic transformation of pan-immune inflammation values; LnSII, natural logarithmic transformation of systemic immune inflammation indices; LnPLR, natural logarithmic transformation of platelet-to-lymphocyte ratios; LnNLR, natural logarithmic transformation of neutrophil-to-lymphocyte ratios; BUN, blood urea nitrogen; eGFR, glomerular filtration rate; UA, uric acid; TP, total protein; TG, triglyceride; TC, cholesterol; LVEF, left ventricular ejection fraction; LVFS, left ventricular fractional shortening; DP, diastolic blood pressure; SP, systolic blood pressure; HR, heart rate.

**Fig. 3.**
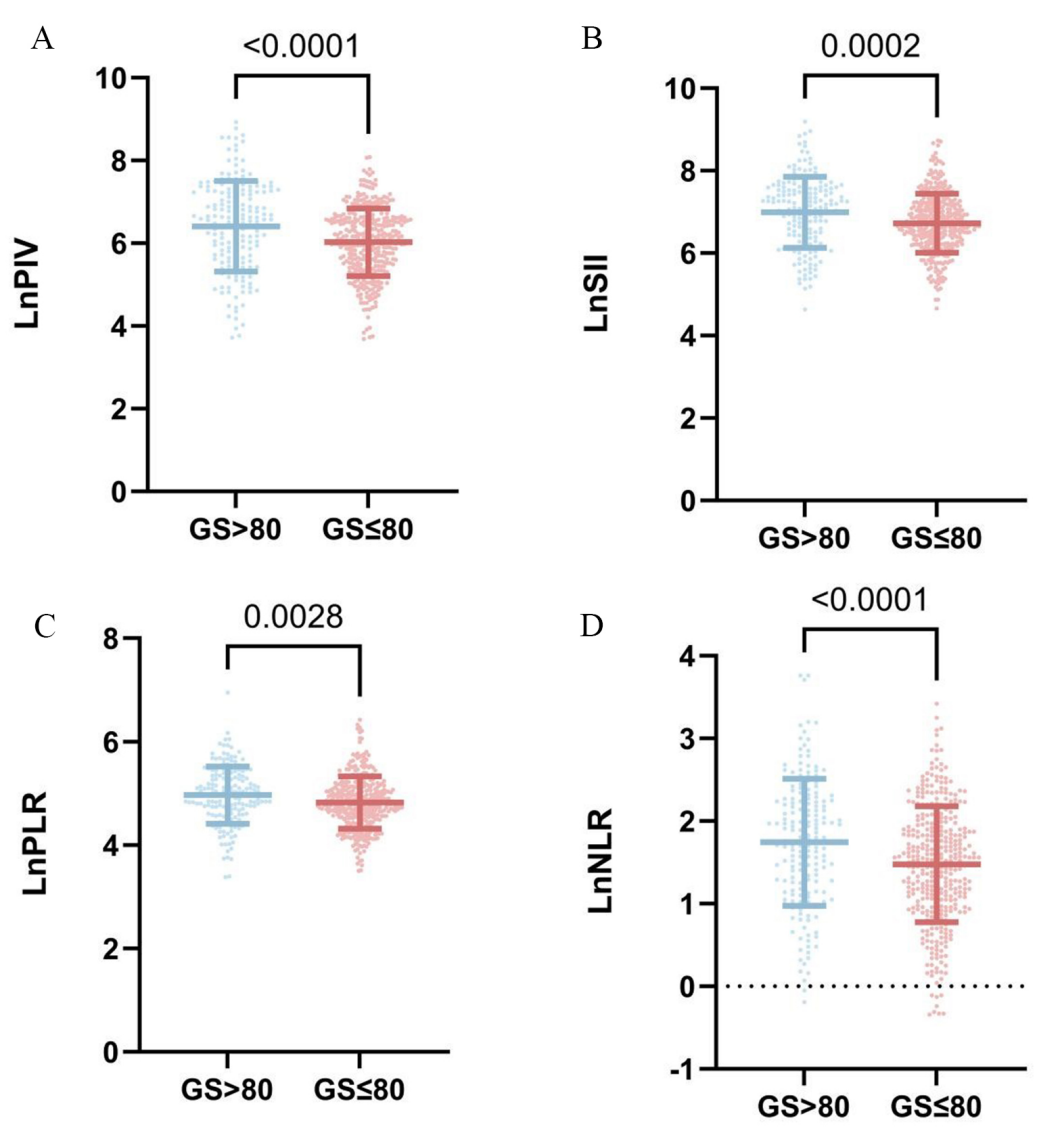
**Comparison of inflammatory markers between the GS >80 and GS ≤80 groups**. (A) LnPIV levels between different GS. (B) LnSII levels between different GS. (C) LnPLR levels between different GS. (D) LnNLR levels between different GS. LnPIV, natural logarithmic transformation of pan-immune-inflammation values; LnSII, natural logarithmic transformation of systemic immune-inflammation indices; LnPLR, natural logarithmic transformation of platelet-to-lymphocyte ratio; LnNLR, natural logarithmic transformation of neutrophil-to-lymphocyte ratio; GS, Gensini score.

The patients were divided into two groups based on GS: the low GS group (GS ≤80) and the high GS group (GS >80). There were 364 patients in the low GS group and 178 patients in the high GS group. Age, DP, HR, neutrophil count, monocyte count, LnPIV, LnSII, LnPLR, LnNLR, BUN, sCr, UA, eGFR, TP, TC, and Killip Class 2–4 were significantly different (*p* < 0.05). The differences in the other indicators were not statistically significant (*p* > 0.05) (Table 3) (Fig. 3).

**Table 3.** **Baseline characteristics of patients in the high Gensini score group (GS >80) versus the nonhigh Gensini score group (GS ≤80)**.

Variables	High GS group (n = 178)	Non-High GS group (n = 364)	*p* value
Age (year)	63.135 ± 13.468	60.074 ± 13.844	0.015
Male [n (%)]	143 (80.3%)	292 (80.2%)	0.974
Smoking [n (%)]	99 (55.6%)	202 (55.5%)	0.978
Hypertension [n (%)]	101 (56.7%)	209 (57.4%)	0.881
Diabetes [n (%)]	51 (28.7%)	92 (25.3%)	0.402
SP (mmHg)	129.401 ± 24.043	126.206 ± 24.184	0.148
DP (mmHg)	79.540 ± 15.542	76.011 ± 15.883	0.015
HR	82.213 ± 20.126	76.772 ± 15.852	<0.001
Neutrophil (×10^9^/L)	7.90 (5.77, 10.82)	6.62 (4.86, 8.94)	<0.001
Lymphocyte (×10^9^/L)	1.36 (1.05, 1.89)	1.49 (1.04, 2.15)	0.112
Monocyte (×10^9^/L)	0.60 (0.40, 0.80)	0.50 (0.40, 0.70)	0.003
Platelet (×10^9^/L)	189.00 (157.00, 230.75)	196.50 (151.25, 241.00)	0.498
LnPIV	6.409 ± 1.094	6.026 ± 0.818	<0.001
LnSII	6.990 ± 0.863	6.727 ± 0.715	<0.001
LnPLR	4.965 ± 0.552	4.822 ± 0.504	0.003
LnNLR	1.743 ± 0.768	1.477 ± 0.702	<0.001
BUN (mmol/L)	5.93 (4.79, 7.36)	5.42 (4.34, 6.70)	<0.001
Creatinine (μmol/L)	72.40 (60.93, 88.00)	71.00 (59.60, 81.38)	0.185
eGFR (mL/(min × 1.73 m^2^))	93.25 (72.59, 105.20)	95.14 (83.16, 105.31)	0.135
UA (mmol/L)	361.46 (295.40, 428.53)	342.35 (291.50, 408.45)	0.119
TP (g/L)	63.13 (60.48, 67.30)	62.55 (59.30, 66.18)	0.043
TG (mmol/L)	1.49 (1.06, 2.10)	1.51 (1.06, 2.22)	0.849
TC (mmol/L)	4.42 (3.88, 5.28)	4.25 (3.59, 4.94)	0.002
LVEF	58.90 (53.75, 62.00)	59.00 (55.25, 64.00)	0.155
LVFS	30.40 (29.00, 33.00)	30.40 (29.00, 33.00)	0.611
Killip Class 2–4 [n (%)]	66 (37.1%)	92 (25.3%)	0.005

Continuous variables are expressed as the mean ± SD, and categorical variables are expressed as percentages. The PIV, SII, PLR, and NLR are skewed data, and the natural logarithmic transformation is used for normally distributed data. SD, standard deviation; GS, Gensini score; LnPIV, natural logarithmic transformation of pan-immune inflammation values; LnSII, natural logarithmic transformation of systemic immune inflammation indices; LnPLR, natural logarithmic transformation of platelet-to-lymphocyte ratios; LnNLR, natural logarithmic transformation of neutrophil-to-lymphocyte ratios; BUN, blood urea nitrogen; eGFR, glomerular filtration rate; UA, uric acid; TP, total protein; TG, triglyceride; TC, cholesterol; LVEF, left ventricular ejection fraction; LVFS, left ventricular fractional shortening; DP, diastolic blood pressure; SP, systolic blood pressure; HR, heart rate.

### 3.2 Univariate and Multivariate Logistic Regression Analysis

Univariate logistic regression analysis showed that age, LnPIV, LnSII, LnPLR, LnNLR, BUN, sCr, UA, neutrophils, and GS were risk factors for the development of MACE after PCI in patients with AMI (*p* < 0.05) (Table 4). The variables that showed relevance in the univariate logistic regression were incorporated into the multivariate logistic regression study together with findings from earlier research. The findings showed that in AMI patients undergoing PCI, neutrophil count, LnPIV, LnSII, LnPLR, and LnNLR continued to be independent risk factors for the development of MACE. (LnPIV odds ratio (OR): 3.520, 95% CI: 2.056–6.026, *p* < 0.001) (LnSII OR: 1.774, 95% CI: 1.348–2.336, *p* < 0.001) (LnPLR OR: 15.783, 95% CI: 1.489–167.253, *p* = 0.022) (LnNLR OR: 30.675, 95% CI: 2.101–447.876, *p* = 0.012) (*p* < 0.05) (Table 4).

**Table 4.** **Univariate logistic regression analysis of major in-hospital adverse cardiovascular events and multivariate logistic regression analysis of selected variables**.

Variables	Univariate logistic regression analysis		Multivariate logistic regression analysis	
	OR (95% CI)	*p* value	OR (95% CI)	*p* value
Age (year)	1.024 (1.010, 1.038)	<0.001	1.003 (0.977, 1.029)	0.838
Male [n (%)]	0.841 (0.536, 1.319)	0.450		
Smoking [n (%)]	0.805 (0.558, 1.162)	0.246		
DP (mmHg)	0.987 (0.975, 0.999)	0.036	1.001 (0.987, 1.014)	0.929
LnPIV	2.309 (1.830, 2.915)	<0.001	2.159 (1.676, 2.782)	<0.001
LnSII	2.019 (1.562, 2.609)	<0.001	1.774 (1.348, 2.336)	<0.001
LnPLR	1.736 (1.215, 2.480)	0.002	1.548 (1.052, 2.278)	0.027
LnNLR	2.069 (1.576, 2.716)	<0.001	1.669 (1.238, 2.249)	<0.001
BUN (mmol/L)	1.284 (1.178, 1.398)	<0.001	1.116 (0.992, 1.257)	0.068
Creatinine (μmol/L)	1.016 (1.010, 1.023)	<0.001	1.001 (0.986, 1.016)	0.917
eGFR(mL/(min × 1.73 m^2^))	0.978 (0.970, 0.986)	<0.001	0.992 (0.967, 1.018)	0.558
UA (mmol/L)	1.003 (1.001, 1.004)	0.003	1.000 (0.998, 1.003)	0.766
TC (mmol/L)	0.791 (0.661, 0.947)	0.011	0.784 (0.638, 0.962)	0.020
Gensini Score	1.009 (1.003, 1.015)	0.005	1.001 (0.994, 1.008)	0.734
LVEF	1.001 (0.995, 1.007)	0.738		
LVFS	0.951 (0.918, 0.986)	0.006	0.973 (0.937, 1.011)	0.166

The covariance test suggested that neutrophils, monocytes, lymphocytes, and platelets have strong covariance and may interfere with the accuracy of the logistic regression results; therefore, these indices were excluded from the logistic regression. LnPIV, natural logarithmic transformation of pan-immune-inflammation values; LnSII, natural logarithmic transformation of systemic immune-inflammation indices; LnPLR, natural logarithmic transformation of platelet-to-lymphocyte ratio; LnNLR, natural logarithmic transformation of neutrophil-to-lymphocyte ratio; BUN, blood urea nitrogen; UA, uric acid; TC, cholesterol; LVEF, left ventricular ejection fraction; LVFS, left ventricular fractional shortening; DP, diastolic blood pressure; eGFR, glomerular filtration rate; OR, odds ratio.

GS was split into two groups based on the results of the risk factor analysis: a low GS group (GS ≤80) and a high GS group (GS >80). We performed a univariate logistic regression analysis taking smoking history, sex, and age into account. The results of the research showed that risk variables for the development of GS were age, DP, monocyte count, LnPIV, LnSII, LnPLR, LnNLR, BUN, sCr, UA, and TC. In addition, preventive factors against the incidence of GS were eGFR and LVEF. (eGFR: OR = 0.989, 95% CI: 0.982–0.997, LVEF: OR = 0.980, 95% CI: 0.961–0.999) (*p* < 0.05) (Table 5). The variables that showed relevance in the univariate logistic regression were incorporated into the multivariate logistic regression study together with findings from earlier research. The findings showed that BUN, TC, LnPIV, LnSII, LnPLR, LnNLR, DP, and monocyte count continued to be separate risk factors for the development of GS. (LnPIV OR: 1.917, 95% CI: 1.192–3.084, *p* = 0.007) (LnSII OR: 1.516, 95% CI: 1.184–1.941, *p* < 0.001) (LnPLR OR: 1.876, 95% CI: 0.859–4.098, *p* = 0.114) (LnNLR OR: 2.032, 95% CI: 1.038–3.979, *p* = 0.038) (*p* < 0.05) (Table 5).

**Table 5.** **Univariate logistic regression analyses for GS >80 and GS ≤80 and multivariate logistic regression analyses with selected variables**.

Variables	Univariate logistic regression analysis		Multivariate logistic regression analysis	
	OR (95% CI)	*p* value	OR (95% CI)	*p* value
Age (year)	1.016 (1.003, 1.030)	0.016	1.018 (0.995, 1.042)	0.126
Male [n (%)]	1.007 (0.642, 1.581)	0.974		
Smoking [n (%)]	1.005 (0.701, 1.442)	0.978		
DP (mmHg)	1.014 (1.003, 1.026)	0.016	1.019 (1.007, 1.032)	0.002
LnPIV	1.580 (1.289, 1.937)	<0.001	1.526 (1.237, 1.882)	<0.001
LnSII	1.565 (1.231, 1.989)	<0.001	1.516 (1.184, 1.941)	<0.001
LnPLR	1.695 (1.194, 2.404)	0.003	1.638 (1.138, 2.359)	0.008
LnNLR	1.668 (1.291, 2.155)	<0.001	1.617 (1.240, 2.109)	<0.001
eGFR(mL/(min × 1.73 m^2^))	0.989 (0.982, 0.997)	0.007	1.004 (0.983, 1.025)	0.716
Creatinine (μmol/L)	1.006 (1.000, 1.011)	0.033	1.000 (0.990, 1.011)	0.967
BUN (mmol/L)	1.153 (1.070, 1.243)	<0.001	1.159 (1.037, 1.295)	0.010
UA (mmol/L)	1.002 (1.000, 1.004)	0.036	1.001 (0.999, 1.003)	0.452
TC (mmol/L)	1.293 (1.090, 1.533)	0.003	1.366 (1.134, 1.645)	0.001
LVEF	0.980 (0.961, 0.999)	0.036	0.986 (0.966, 1.006)	0.178
LVFS	0.979 (0.946, 1.014)	0.238		

The covariance test suggested that neutrophils, monocytes, lymphocytes, and platelets have strong covariance and may interfere with the accuracy of the logistic regression results; therefore, these indices were excluded from the logistic regression. LnPIV, natural logarithmic transformation of pan-immune-inflammation values; LnSII, natural logarithmic transformation of systemic immune-inflammation indices; LnPLR, natural logarithmic transformation of platelet-to-lymphocyte ratio; LnNLR, natural logarithmic transformation of neutrophil-to-lymphocyte ratio; BUN, blood urea nitrogen; UA, uric acid; TC, cholesterol; LVEF, left ventricular ejection fraction; LVFS, left ventricular fractional shortening; DP, diastolic blood pressure; eGFR, glomerular filtration rate; OR, odds ratio.

### 3.3 Relationship between PIV Level and MACE

We divided the patients into four groups according to PIV quartile values (LnPIV ≤5.52; 5.52 < LnPIV ≤ 6.18; 6.18 < LnPIV ≤ 6.76; LnPIV ≥6.76) to further illustrate the connection between PIV level and MACE. We discovered that there was a substantial correlation between the level of PIV and the chance of having a MACE. The OR for MACE in the highest quartile in Model 1, without variable adjustment, was 6.51 (95% CI: 3.68–11.49, *p* < 0.001). When covariates were taken into account, Model 3 produced an OR of 6.89 (95% CI: 3.56–13.32, *p* < 0.001) for the highest MACE quartile (Table 6). Ultimately, a statistically significant trend showing an increasing probability of MACE with a larger PIV was revealed by the trend analysis results (OR: 1.86, 95% CI: 1.51–2.29, *p* < 0.001) (Table 6).

**Table 6.** **Trend analysis of different levels of PIV and the occurrence of MACE**.

		Model 1		Model 2		Model 3	
		OR (95% CI)	*p* value	OR (95% CI)	*p* value	OR (95% CI)	*p* value
LnPIV		2.31 (1.83, 2.91)	<0.0001	2.31 (1.83, 2.93)	<0.0001	2.27 (1.73, 2.98)	<0.0001
LnPIV Quartile							
	LnPIV ≤5.52	1.0		1.0		1.0	
	5.52 < LnPIV ≤ 6.18	1.45 (0.79, 2.68)	0.2299	1.47 (0.79, 2.73)	0.2211	1.81 (0.93, 3.51)	0.0808
	6.18 < LnPIV ≤ 6.76	2.01 (1.11, 3.63)	0.0204	2.03 (1.12, 3.68)	0.0204	2.35 (1.22, 4.51)	0.0104
	LnPIV ≥6.76	6.51 (3.68, 11.49)	<0.0001	6.78 (3.80, 12.10)	<0.0001	6.89 (3.56, 13.32)	<0.0001
LnPIV for trend		1.88 (1.57, 2.25)	<0.0001	1.90 (1.58, 2.28)	<0.0001	1.86 (1.51, 2.29)	<0.0001

Model 1: No adjustment model. Model 2: Adjusted for age, male, smoking, diabetes, hypertension. Model 3: Adjusted for age, male, smoking, diabetes, hypertension, systolic perssure, diastolic perssure, heart rate, BUN, creatinine, eGFR, UA, TP, TG, TC, Gensiniscore, LVEF, LVFS, and Killip class2–4. LnPIV, natural logarithmic transformation of pan-immune-inflammation values; MACE, major adverse cardiovascular events; BUN, blood urea nitrogen; UA, uric acid; TC, cholesterol; LVEF, left ventricular ejection fraction; LVFS, left ventricular fractional shortening; eGFR, glomerular filtration rate; PIV, Pan-immune-inflammation value; TP, total protein; TG, triglyceride.

### 3.4 Relationship between PIV Level and GS

To further demonstrate the relationship between PIV level and GS, we categorized patients into four groups based on PIV quartile values (LnPIV ≤5.52; 5.52 < LnPIV ≤ 6.18; 6.18 < LnPIV ≤ 6.76; LnPIV ≥6.76). PIV was significantly associated with the risk of a higher coronary disease burden as reflected by the GS. The patients did not develop a GS, they have an underlying GS. In Model 1, without adjustment for variables, the OR for GS in the highest quartile was 2.69 (95% CI: 1.63–4.44, *p* < 0.001). Model 3 adjusted for confounders resulted in an OR of 2.69 (95% CI: 1.56–4.62, *p* < 0.001) for the highest quartile of GS (Table 7). Finally, the results of the trend analysis revealed a statistically significant trend indicating an increasing probability of GS with a higher PIV (OR: 1.42, 95% CI: 1.19–1.70, *p* < 0.001) (Table 7).

**Table 7.** **Trend analysis of different levels of PIV with GS**.

		Model 1		Model 2		Model 3	
		OR (95% CI)	*p* value	OR (95% CI)	*p* value	OR (95% CI)	*p* value
LnPIV		1.58 (1.29, 1.94)	<0.0001	1.56 (1.27, 1.91)	<0.0001	1.58 (1.27, 1.96)	<0.0001
LnPIV Quartile							
	LnPIV ≤5.52	1.0		1.0		1.0	
	5.52 < LnPIV ≤ 6.18	0.73 (0.42, 1.26)	0.2526	0.72 (0.42, 1.25)	0.2473	0.69 (0.38, 1.24)	0.2137
	6.18 < LnPIV ≤ 6.76	0.93 (0.55, 1.58)	0.7867	0.93 (0.54, 1.60)	0.8035	0.89 (0.50, 1.60)	0.7020
	LnPIV ≥6.76	2.69 (1.63, 4.44)	0.0001	2.59 (1.56, 4.30)	0.0002	2.69 (1.56, 4.62)	0.0003
LnPIV for trend		1.41 (1.20, 1.67)	<0.0001	1.40 (1.19, 1.65)	<0.0001	1.42 (1.19, 1.70)	<0.0001

Model 1: No adjustment model. Model 2: Adjusted for age, male, smoking, diabetes, hypertension. Model 3: Adjusted for age, male, smoking, diabetes, hypertension, systolic perssure, diastolic perssure, heart rate, BUN, creatinine, eGFR, UA, TP, TG, TC, LVEF, LVFS, and Killip class2–4. LnPIV, natural logarithmic transformation of pan-immune-inflammation values; GS, gensini score; BUN, blood urea nitrogen; UA, uric acid; TC, cholesterol; LVEF, left ventricular ejection fraction; LVFS, left ventricular fractional shortening; eGFR, glomerular filtration rate; PIV, Pan-immune-inflammation value; TP, total protein; TG, triglyceride.

### 3.5 Subgroup Analysis

**Fig. 4.**
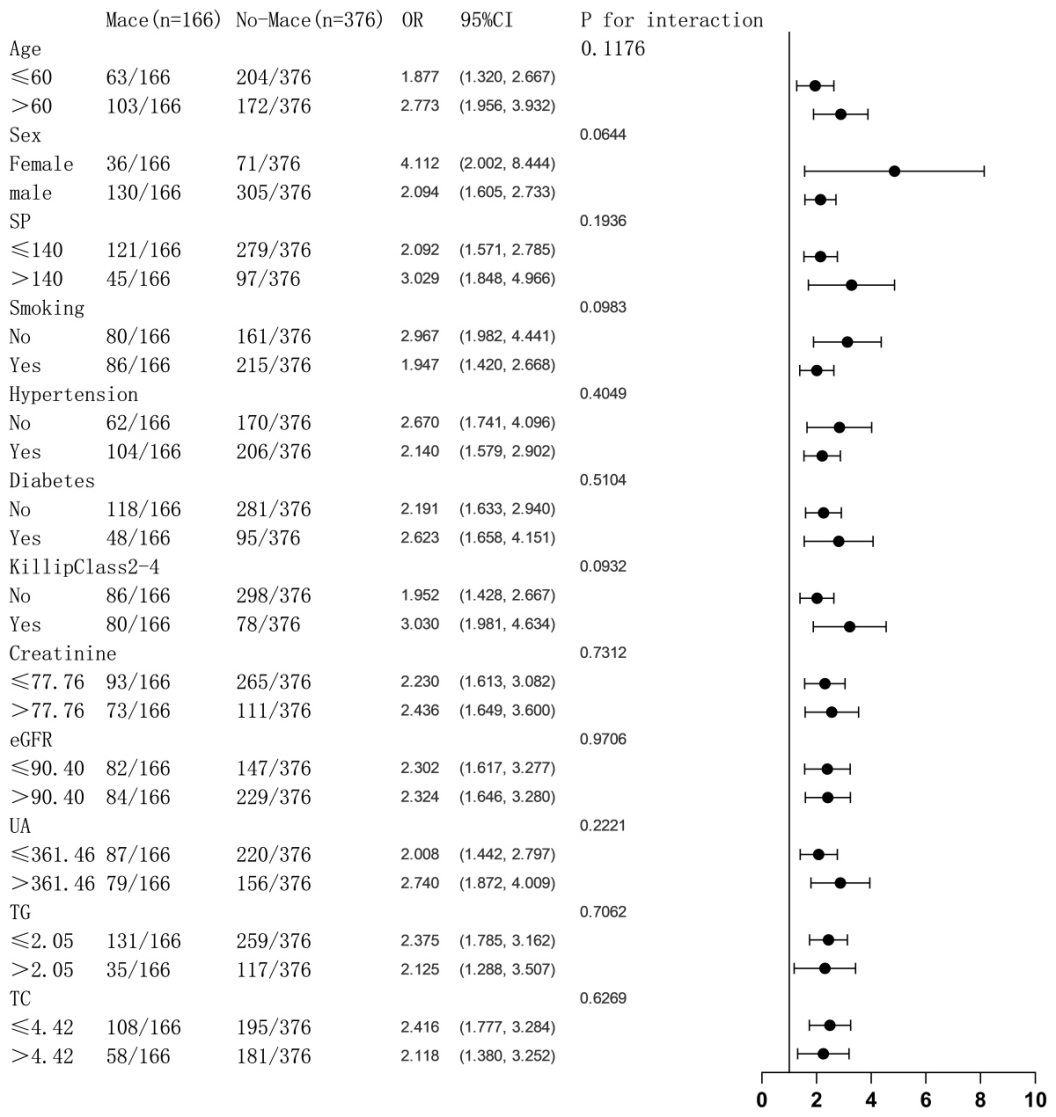
**Variables influencing the connection between PIV and MACE are examined in a categorized logistic regression analysis model**. The above models were adjusted for age, sex, SP, smoking status, diabetes mellitus status, hypertension status, Killip classification 2–4, sCr level, eGFR, UA level, TG level, and TC level. sCr, serum creatinine; UA, uric acid; eGFR, glomerular filtration rate; TG, triglyceride; TC, cholesterol; PIV, Pan-immune-inflammation value; MACE, major adverse cardiovascular events; SP, systolic blood pressure.

To investigate whether the levels of other variables affect the correlation between PIV and MACE, we divided the variables between the models into two groups: age (≤60 vs >60), gender (female vs male), SP (≤140 vs >140), smoking status, hypertension status, diabetes mellitus status, Killip classification 2–4, sCr level (≤77.76 vs >77.76), eGFR (≤90.40 vs >90.40), UA level (≤361.46 vs >361.46), TG level (≤2.05 vs >2.05), and TC level (≤4.42 vs >4.42). We aimed to determine whether the differences in the combined effect sizes of the subgroups were statistically significant. Subgroup analyses showed no statistically significant interaction test results for the PIV and MACE-related effects of the stratification factors in the model (*p* > 0.05 for all interactions) (Fig. 4). There was no interaction between the grouping factors and the combined effect sizes. Additionally, there was no effect of the stratification factors on the correlation between PIV and MACE (Fig. 4).

**Fig. 5.**
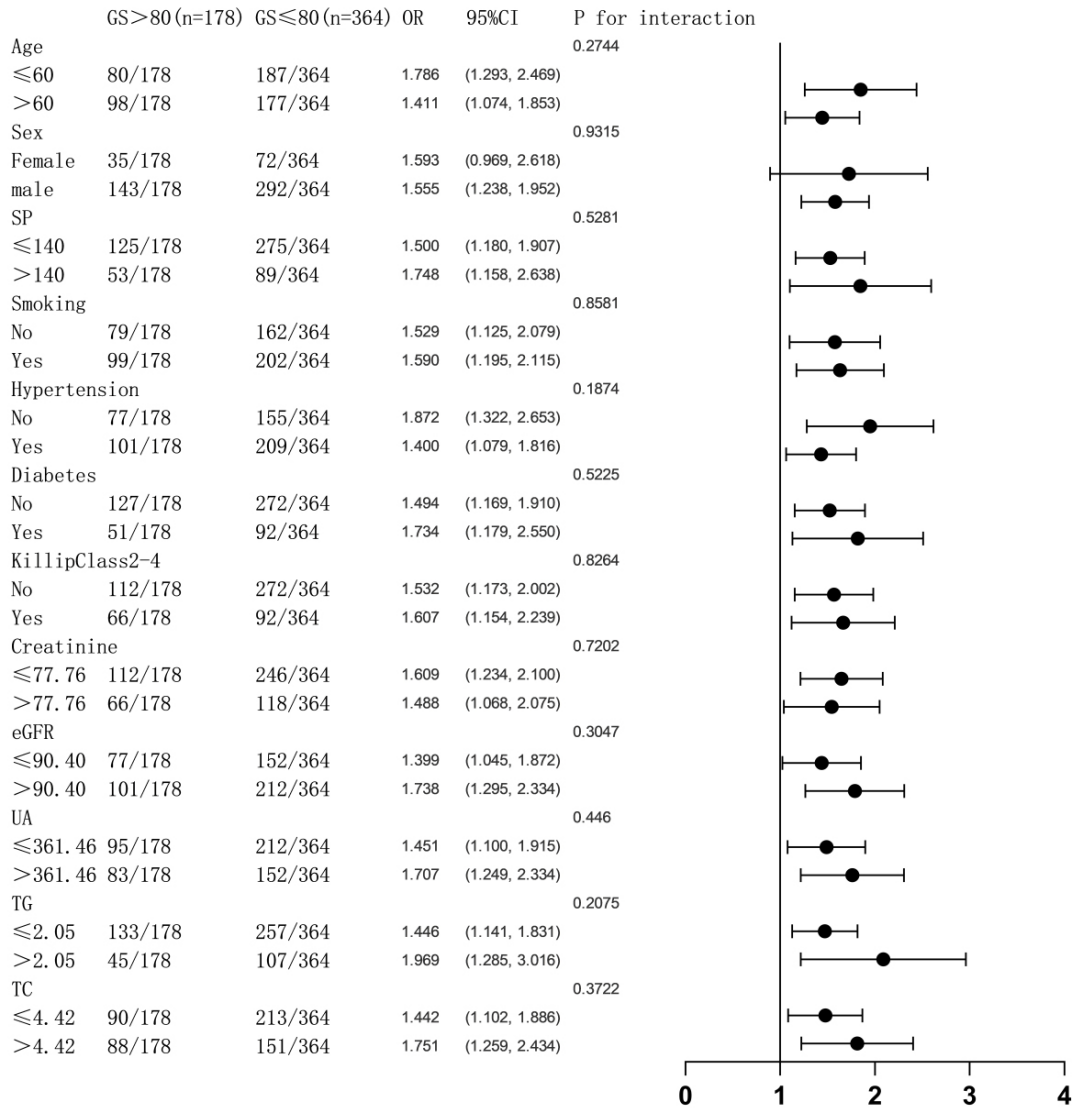
**Variables influencing the connection between PIV and GS are examined in a categorized logistic regression analysis model (GS >80)**. The above models were adjusted for age, sex, SP, smoking status, diabetes mellitus status, hypertension status, Killip classification 2–4, sCr level, eGFR, UA level, TG level, and TC level. sCr, serum creatinine; UA, uric acid; eGFR, glomerular filtration rate; TG, triglyceride; TC, cholesterol; PIV, Pan-immune-inflammation value; GS, Gensini score; SP, systolic blood pressure.

To determine whether the levels of other variables influenced the correlation between PIV and GS, we conducted an additional set of subgroup analyses. The results of the analysis revealed age ≤60 vs >60 years, sex ≤140 vs >140, smoking status, hypertension status, diabetes mellitus status, Killip classification 2–4, sCr level ≤77.76 vs >77.76, eGFR ≤90.40 vs >90.40, UA level ≤361.46 vs >361.46, TG level ≤2.05 vs >2.05, and TC level ≤4.42 vs >4.42. The results of the interaction test for the effects related to PIV and GS were not statistically significant (*p* > 0.05 for all interactions) (Fig. 5). There was no interaction between the grouping factors and the combined effect sizes. Additionally, stratification factors did not affect the correlation between PIV and GS (Fig. 5).

### 3.6 ROC Curve Analysis

**Fig. 6.**
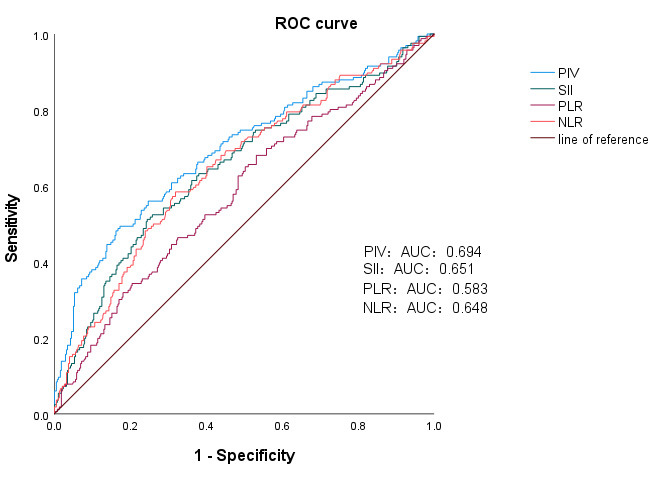
**ROC curves of PIV, the SII, the PLR, and the NLR for predicting the risk of MACE during hospitalization in STEMI patients**. PIV, SII, PLR, and NLR Predictive Levels of MACE Occurrence. ROC curve, receiver operating characteristic curve; AUC, area under the curve; MACE, major adverse cardiovascular events; STEMI, ST-segment elevation myocardial infarction; PIV, pan-immune-inflammation value; SII, systemic immune-inflammation index; PLR, platelet-to-lymphocyte ratio; NLR, neutrophil-to-lymphocyte ratio.

ROC curve analysis was used to compare the predictive performance of PIV with that of the traditional inflammatory indicators SII, PLR, and NLR for in-hospital MACE after initial PCI in AMI patients. According to the ROC curve analysis, the area under the curve (AUC) of PIV was 0.694, with a critical value of 793.755, a specificity of 82.7%, and a sensitivity of 49.4%. The AUC of the SII was 0.651, with a critical value of 1308.285, a specificity of 75.50%, and a sensitivity of 51.20%. The AUC of the PLR was 0.583, with a critical value of 118.06, a specificity of 46.80%, and a sensitivity of 68.10%. The NLR had an AUC of 0.648, a critical value of 5.805, a specificity of 68.10%, and a sensitivity of 58.40% (Table 8) (Fig. 6).

**Table 8.** **ROC curves of the PIV, SII, PLR, and NLR for predicting MACE**.

Variables	AUC	Sensitivity	Specificity	Cut-off value (×10^9^/L)	*p* value
PIV	0.694	0.494	0.827	793.755	<0.001
SII	0.651	0.512	0.755	1308.285	<0.001
PLR	0.583	0.681	0.468	118.06	0.002
NLR	0.648	0.584	0.681	5.805	<0.001

ROC, receiver operating characteristic; AUC, area under the curve; PIV, pan-immune-inflammation value; SII, systemic immune-inflammation index; PLR, platelet-to-lymphocyte ratio; NLR, neutrophil-to-lymphocyte ratio; MACE, major adverse cardiovascular event.

**Fig. 7.**
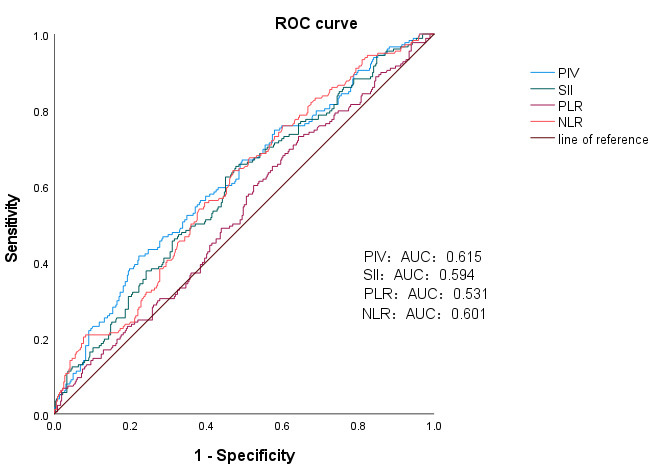
**ROC curves of PIV, the SII, the PLR, and the NLR for predicting the risk of GS >80 during hospitalization in STEMI patients**. PIV, SII, PLR, and NLR Predictive Levels of GS Occurrence. ROC curve, receiver operating characteristic curve; AUC, area under the curve; GS, Gensini score; STEMI, ST-segment elevation myocardial infarction; PIV, pan-immune-inflammation value; SII, systemic immune-inflammation index; PLR, platelet-to-lymphocyte ratio; NLR, neutral neutrophil-to-lymphocyte ratio.

Compared with the traditional inflammatory indicators SII, PLR, and NLR, PIV had an AUC of 0.615, a critical value of 771.75, a specificity of 77.70%, and a sensitivity of 41.60% for predicting high GS. The SII had an AUC of 0.594, a critical value of 828.355, a specificity of 52.20%, and a sensitivity of 65.20%. The PLR had an AUC of 0.531, a critical value of 106.73, a specificity of 35.70%, and a sensitivity of 73.00%. The NLR had an AUC of 0.601, a critical value of 4.735, a specificity of 53.00%, and a sensitivity of 64.00% (Table 9) (Fig. 7).

**Table 9.** **ROC curves of the PIV, SII, PLR, and NLR for predicting GS**.

Variables	AUC	Sensitivity	Specificity	Cut-off value (×10^9^/L)	*p* value
PIV	0.615	0.416	0.777	771.75	<0.001
SII	0.594	0.652	0.522	828.355	<0.001
PLR	0.531	0.73	0.357	106.73	0.234
NLR	0.601	0.64	0.53	4.735	<0.001

ROC, receiver operating characteristic; AUC, area under the curve; PIV, pan-immune-inflammation value; SII, systemic immune-inflammation index; PLR, platelet-to-lymphocyte ratio; NLR, neutrophil-to-lymphocyte ratio; GS, Gensini score.

It is clear from the results that the AUC of PIV is greater than that of other traditional inflammatory indicators, such as the SII, PLR, and NLR. The PIV demonstrated better predictive performance for in-hospital MACE after initial PCI and for coronary stenosis in patients with AMI.

### 3.7 Correlation between PIV, SII, PLR, NLR, and GS

**Fig. 8.**
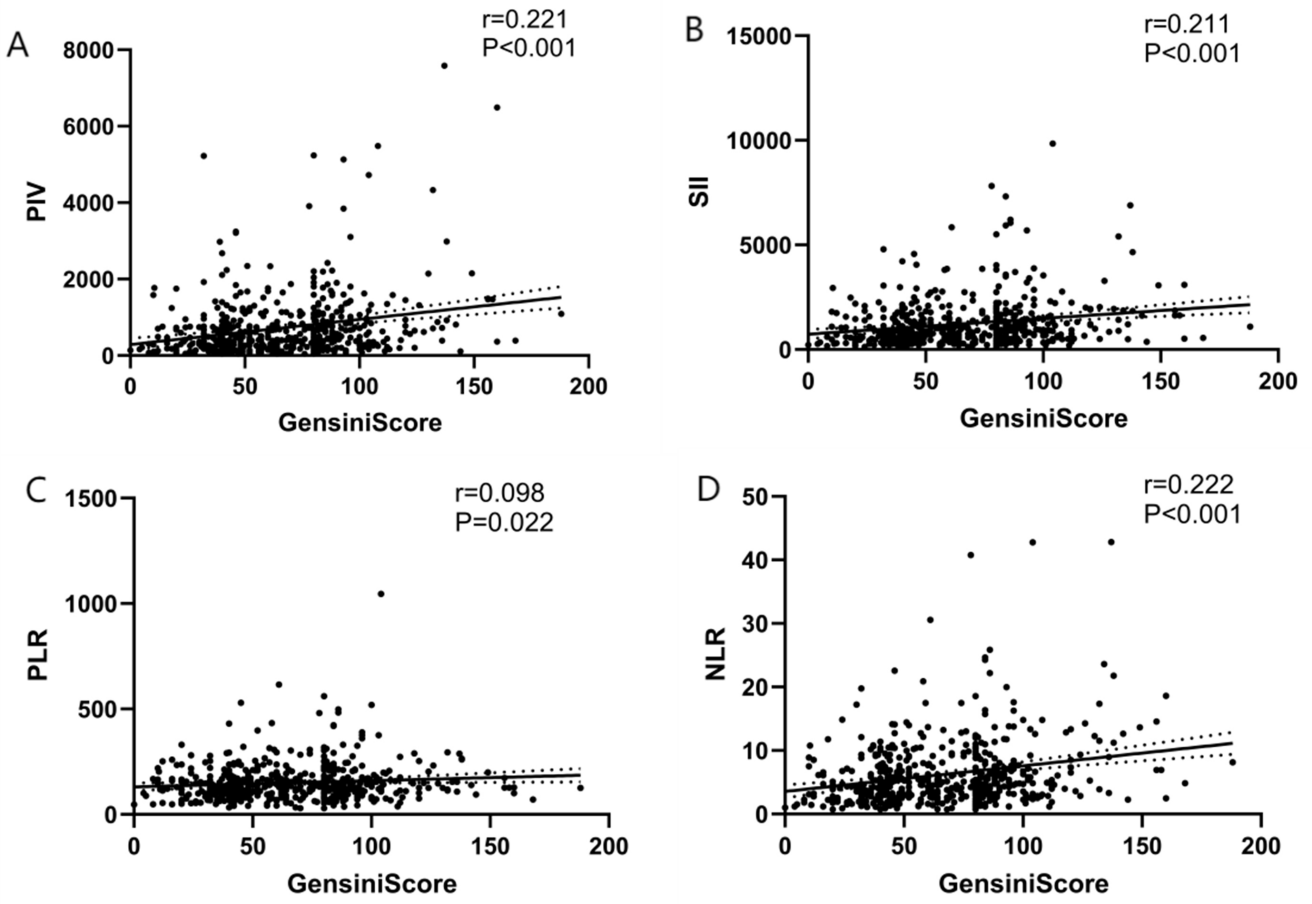
**Correlations of PIV, the SII, the PLR, and the NLR with the Gensini score (Spearman correlation analysis)**. (A) Correlation between PIV and GS. (B) Correlation between SII and GS. (C) Correlation between PLR and GS. (D) Correlation between NLR and GS. PIV, pan-immune-inflammation value; SII, systemic immune-inflammation index; PLR, platelet-to-lymphocyte ratio; NLR, neutrophil-to-lymphocyte ratio; GS, Gensini score.

To further explore the correlation between PIV, the SII, the PLR, the NLR, and the GS, we conducted a Spearman correlation analysis. The results indicated significant correlations between PIV, the SII, and the NLR with the GS (PIV: r = 0.221, *p* < 0.001; SII: r = 0.211, *p* < 0.001; NLR: r = 0.222, *p* < 0.001), and the PLR showed a weaker correlation with the GS (PLR: r = 0.098, *p* < 0.022) (Table 10) (Fig. 8).

**Table 10.** **Correlations of PIV, the SII, the PLR, and the NLR with GS**.

Variables	r	*p* value
PIV	0.221	<0.001
SII	0.211	<0.001
PLR	0.098	0.022
NLR	0.222	<0.001

Spearman correlation analysis. GS, Gensini score; PIV, pan-immune-inflammation value; SII, systemic immune-inflammation index; PLR, platelet-to-lymphocyte ratio; NLR, neutrophil-to-lymphocyte ratio; r, correlation coefficient.

These findings suggest that PIV, the SII, and the NLR correlate with the degree of coronary artery stenosis, as reflected by GS, and that the difference in correlation between PIV, the SII, the NLR, and GS is not significant.

## 4. Discussion

The following are the study’s primary findings: Compared to SII, PLR, or NLR, PIV provides a higher predictive value for the incidence of MACE following PCI in hospitalized STEMI patients. Second, in terms of predicting the degree of coronary artery stenosis, PIV seems to be more accurate than SII, PLR, and NLR. To the best of our knowledge, there are currently no extensive studies regarding the predictive value of PIV for the occurrence of short-term MACE and the severity of coronary stenosis based on the GS during post-PCI hospitalization in STEMI patients.

The basic pathogenic mechanism of AMI is the narrowing of the lumen and myocardial ischemia due to coronary atherosclerosis, and collateral circulation has not yet been established in time, which leads to the obstruction of the heart’s blood supply channels, resulting in myocardial ischemia, hypoxia, myocardial injury, and ultimately the development of myocardial necrosis. The role of adipokines in AMI has been well studied. Adipokines regulate the development and progression of AMI, and adipokines such as IL-10, omentin-1, andghrelininhibit AMI-induced inflammatory responses [22]. Also, along with adipokines, inflammatory processes, and thrombosis playan importantrole in the development and progression of AMI. It hasbeen shownthat high leukocyte levels are associated with the incidence of STEMI. Different subtypes of leukocytes (neutrophils, lymphocytes, monocytes) regulate the inflammatory process in STEMI.

Neutrophils are the most abundant type of leukocyte, and they are the first inflammatory cells involved in plaque formation. After AMI, due to stimulation by extracellular physicochemical factors, neutrophil nuclear chromatin loses its normal morphology, and enzymes in intracellular vesicles are combined, followed by the rapid release of various enzymes and cytokines from neutrophils, which results in the formation of extracellular neutrophil extracellular traps (NETs) [23]. The activation of other immune system cells triggers and potentiates inflammatory responses, and the NET burden is positively correlated with myocardial infarct size [23, 24, 25]. In addition, in the early stages of myocardial infarction, monocytes, through chemokines and cytokines, can recognize damage-associated molecular patterns (DAMPs) released by dead cardiomyocytes, thereby triggering a pathological inflammatory response. In the pathological state, monocytes are transformed into macrophages, whose AXL receptor tyrosine kinases cause cardiac inflammation after reperfusion due to myocardial infarction [26, 27]. Previous studies have shown that monocytes are heterogeneous and are classified into classic, intermediate, and atypical monocytes depending on their surface receptors, with intermediate monocytes (IMs) exhibiting increased abundance due to stimulation by NETs and decreased CX3CR1 expression, which may lead to decreased transport of atypical monocytes to ischemic tissues and impaired myocardial healing [25]. On the other hand, lymphocytes reflect a calm and regulated inflammatory process. Lymphocytes impede the progression of atherosclerosis [28]. Lower lymphocyte counts correlate with MACE in STEMI patients [29]. In addition, platelets play an important role in atherosclerosis, and their activation promotes inflammation and thrombosis [30, 31, 32]. Extracellular NETs activate the immune system, inducing platelet activation and initiating coagulation, which promotes thrombosis and AMI occurrence [23].

The four cell counts mentioned above have been widely studied in the literature as markers due to their low cost and ease of access. In recent years, composite inflammatory indicators have been valued by researchers for reflecting the inflammatory state more comprehensively. The NLR is a measure of the ratio of neutrophils to lymphocytes. Because the number of lymphocytes and neutrophils is correlated with the overall level of inflammation in the body, the NLR can be used to predict the prognosis of a number of diseases, including septicemia, ulcerative colitis, and colorectal cancer, through determining imbalances between the two cell populations [33, 34, 35]. The PLR has been thoroughly researched in relation to rheumatic disorders and AMI in older adults. Because it combines the properties of lymphocytes and platelets, it is thought to be a signal of inflammation [36, 37]. Nonetheless, there appears to be a considerable correlation between the PLR and the degree of persistent inflammation. Atherosclerosis and microvascular damage brought on by persistent inflammation raise mortality risk. The PLR is a predictor of long-term mortality in individuals with ACS, according to several studies [10]. The PLR and NLR are helpful and strong independent predictors of MACE in patients with STEMI and can be useful in predicting prognosis in patients with STEMI, according to research reporting on these markers in patients with STEMI [11, 14]. In addition, the SII is the ratio of platelets combined with neutrophils to lymphocytes. In addition, it was recently reported that the SII was associated with MACE in STEMI patients [15, 38], and its predictive efficacy for MACE was superior to that of the PLR and NLR [35, 36], which is similar to the results we observed in the present study. In previous studies, it has been shown that the inflammatory marker SII calculated from three inflammatory parameters has a greater predictive value for MACE occurrence in STEMI patients than the inflammatory markers PLR and NLR calculated from two parameters. In consideration of the previous research, the current study examined if adding more inflammatory parameters would improve the composite biomarkers’ predictive value. As a result, PIV—a unique inflammatory indicator—was presented in this work as a new marker. It was computed using 4 inflammatory cells. The PIV is the ratio of monocytes to lymphocytes, together with platelets and neutrophils. In this study, PIV provided a comprehensive definition of the state of systemic inflammation and immune system activation in STEMI patients by combining neutrophils, platelets, lymphocytes, and monocytes. PIV has been shown to have predictive significance in cases of breast, colorectal, esophageal, and oral squamous cell carcinoma in earlier research [16, 17, 18, 39]. In addition, in STEMI patients, PIV is linked to decreased coronary flow (ICF). There is a substantial correlation between high PIV levels and a higher risk of ICF following PCI [20]. In patients with STEMI, PIV at 12 hours after PCI may be a more reliable and economical indicator of a poor long-term prognosis [40]. The delayed filling of coronary end-vessels with contrast agents in the presence of normal or nearly normal epicardial coronary arteries is known as the coronary slow-flow phenomenon (CSFP). Individuals who have elevated PIV levels are more susceptible to CSFP [21]. Recently, it was published that in patients with non-STEMI, the PIV was substantially correlated with a high syntax score and the severity of CAD [41]. The prognostic efficacy of PIV for the onset of MACE and the severity of coronary stenosis in patients hospitalized with AMI following PCI, however, has not been the subject of many investigations.

According to the clinical observations, there was a significant negative correlation between the occurrence of MACE and patients’ quality of life. In addition, for some patients with comorbid renal failure and tumors, PCI is not suitable for resolving coronary stenosis. These patients have a significantly greater risk of MACE. In this situation, PIV is anticipated to help anticipate the incidence of MACE and the severity of coronary stenosis since it is a quick, precise, affordable, and readily available indicator. The purpose of this study was to evaluate the predictive usefulness of PIV in hospitalized STEMI patients for the occurrence of MACE following PCI against that of other inflammatory markers. The results of the present study showed that the predictive value of PIV for MACE occurrence after PCI in hospitalized STEMI patients was better than that of the SII, PLR, and NLR. Therefore, PIV may be more efficient in predicting the prognosis of STEMI patients. This study also looked at the forecasting valuable GS for estimating the degree of coronary artery stenosis. Studies found that PIV could be helpful for forecasting the level of coronary stenosis as assessed by GS [42]. Thus, PIV is more accurate than SII, PLR, and NLR in predicting the occurrence of MACE and the extent of coronary stenosis after inpatient PCI in STEMI patients.

## 5. Limitations

This study has several limitations. First, the single-center, retrospective approach raises the possibility of bias, which could restrict how broadly the results can be applied. Subsequent research will employ greater sample sizes and people from various regions. Second, the small number of subjects in this study and its restriction to one region may impair the statistical significance of the findings. Third, the emphasis of this investigation was adverse cardiovascular events that occurred during hospitalization; therefore, prospective studies are necessary to examine the long-term prognostic significance of PIV. While we discovered a correlation between PIV levels and the degree of coronary artery stenosis and MACE in patients, additional confirmation of our findings will require multicenter studies with greater numbers of participants and prospective long-term monitoring of patient populations.

## 6. Conclusions

Our study suggested that PIV may have greater predictive value than SII, PLR, or NLR in terms of the occurrence of MACE and predicting the degree of coronary stenosis after PCI in hospitalized STEMI patients.

## Data Availability

The datasets used and/or analyzed in this study are available upon reasonable request from the corresponding author, Jing Zhang. These data are not publicly available because they contain information that may compromise patient privacy.

## Abbreviations

PIV, pan-immune-inflammation value; LnPIV, natural logarithmic transformation of pan-immune-inflammation value; SII, systemic immune-inflammation index; LnSII, the natural logarithmic transformation of the systemic immune-inflammation index; PLR, platelet-to-lymphocyte ratio; LnPLR, natural logarithmic transformation of platelet-to-lymphocyte ratio; NLR, neutrophil-to-lymphocyte ratio; LnNLR, neutrophil-to-lymphocyte ratio, natural logarithmic transformation; PCI, percutaneous coronary intervention; MACE, major adverse cardiovascular events; STEMI, ST-segment elevation myocardial infarction; ROC curve, receiver operating characteristic curve; GS, Gensini score; OR, odds ratio; CI, confidence interval; AUC, area under the curve; SD, standard deviation; CVD, cardiovascular disease; US, United States; NHANES, National Health and Nutrition Examination Survey; CAD, coronary artery disease; AMI, acute myocardial infarction; CAG, coronary angiography; ACS, acute coronary syndrome; SP, systolic blood pressure; DP, diastolic blood pressure; HR, heart rate; sCr, serum creatinine; BUN, blood urea nitrogen; UA, uric acid; eGFR, glomerular filtration rate; TP, total protein; TG, triglyceride; TC, cholesterol; LVEF, left ventricular ejection fraction; LVFS, left ventricular fraction shortening; NETs, neutrophil extracellular traps; DAMPs, damage-associated molecular patterns; IMs, intermediate monocytes; r, correlation coefficient.
